# Utility of Pentraxin-3 as a biomarker for diagnosis of acute appendicitis: a systematic review and meta-analysis

**DOI:** 10.1007/s00383-022-05149-4

**Published:** 2022-06-15

**Authors:** Sachit Anand, Niklas Pakkasjärvi, Minu Bajpai, Nellai Krishnan, Chandramouli Goswami, Janne S. Suominen, Devendra Kumar Yadav, Prabudh Goel

**Affiliations:** 1 Department of Pediatric Surgery, Kokilaben Dhirubhai Ambani Hospital, Mumbai, India; 2 Department of Pediatric Surgery, Turku University Hospital and University of Turku, Turku, Finland; 3 Department of Pediatric Surgery, Helsinki University Hospital and University of Helsinki, Helsinki, Finland; 4 Department of Pediatric Surgery, All India Institute of Medical Sciences, New Delhi, India

**Keywords:** Pentraxin-3, Acute phase protein, Biomarker, Acute appendicitis, Complicated appendicitis, Perforated appendicitis, Non-specific abdominal pain, Children

## Abstract

**Purpose:**

To systematically summarize all relevant data and to define the current evidence on the utility of Pentraxin-3 (PTX3) as a biomarker for acute appendicitis (AA) in children.

**Methods:**

This review was conducted in accordance with the PRISMA guidelines. PubMed, Embase, Scopus, and Web of Science databases were systematically searched for studies comparing the levels of PTX3 in patients with AA vs healthy controls or non-specific abdominal pain (NSAP). Mean differences were calculated for all outcomes and the inverse variance method was used for weighted mean difference. The methodological quality of the included studies was assessed using the Downs and Black scale.

**Results:**

Five comparative studies were included. Significantly elevated levels of PTX3 in cases with AA vs healthy controls (WMD: 9.56, 95% CI 7.24–11.88, *p* < 0.00001), and patients with AA vs NSAP (WMD: 8.05, 95% CI 6.81–9.29, *p* < 0.00001) were demonstrated. Similarly, in separate meta-analyses, the levels of PTX3 were significantly elevated in children with AA vs healthy controls (WMD: 11.18, 95% CI 10.03–12.34, *p* < 0.00001), and children with AA vs NSAP (WMD: 8.35, 95% CI 6.88–9.82, *p* < 0.00001).

**Conclusions:**

PTX3-levels are elevated in AA, but differentiation between perforated and non-perforated appendicitis demands other methods.

## Introduction

Acute appendicitis (AA) is one of the most common causes of right lower quadrant pain in children [1]. Despite evolving treatment options, its diagnosis remains challenging. Presenting signs and symptoms are often variable and initially subtle, especially in children. Due to this, the clinical diagnosis of AA can be delayed and may lead to increased morbidity and mortality [1]. To overcome this, a number of diagnostic tests are frequently ordered by the clinicians to confirm the diagnosis of AA. These include inflammatory markers, such as WBC, CRP, IL-6, etc., with radiological studies, such as ultrasonography and contrast-enhanced abdominal computed tomography. However, the findings of these adjunctive tests might not support the diagnosis during the initial phases of AA and may cause an inadvertent delay [2].

AA, a suppurative inflammation, causes a rise in several biomarkers notably the pentraxin group of multimeric proteins. As a part of the humoral immune response, the levels of these proteins rise during inflammation [3]. C-reactive protein, a short pentraxin (based on length of N-terminal region), peaks around 48 h of inflammation [4]. Pentraxin-3 (PTX-3) is a long pentraxin and is produced locally at the inflammation site by epithelial, mesenchymal, endothelial and myeloid cells in response to inflammatory cytokines, such as IL-1, TNF-inducible gene, etc. Although CRP and PTX-3 show low plasma concentrations in healthy children, their levels rapidly rise during inflammatory conditions, with CRP reaching its maximum peak at 48 h and PTX-3 in 6 h. This swift increase in PTX-3 is due to its local production by a number of cells and within the granules of the neutrophils [5]. Peak levels of PTX-3 are reached by 6 h, thus, serving as a novel biomarker for early diagnosis of AA [6].

Several scientific reports have been published regarding the use of PTX-3 as a biomarker for the diagnosis of AA; however, a systematic review on this subject is lacking. In the light of the abovementioned information, we conducted a systematic review on the utility of PTX-3 as a novel biomarker for the diagnosis of AA. We hypothesized that the levels of PTX-3 in cases of AA, in both children and adults, are significantly higher as compared to those in healthy controls and cases with non-specific abdominal pain. To our best knowledge, ours is the first study of its kind to focus on this research subject.

## Methods

### Registration and search strategy

The present systematic review was registered in the International prospective register of systematic reviews (PROSPERO) on 07 Jan 2022 (CRD42022296832). The study was conducted as per the Preferred Reporting Items for Systematic Reviews and Meta-Analyses (PRISMA) guidelines [7]. Two investigators, NK and SA, searched the PubMed, Embase, Scopus and Web of science databases systematically. The search terms (Pentraxin-3) AND (appendicitis) were used to identify all papers highlighting the role of PTX-3 as a biomarker for AA. No age filter was applied and the studies including both children and adults were included. Cross citations and articles not included in the above databases were also identified by snowballing and reverse snowballing. The duplicate entries were removed and the remaining articles were screened to select the relevant studies as per the eligibility criteria.

### Eligibility criteria

The inclusion criteria for the present systematic review were: Participants—all patients (of any age) with a clinico-radiologic diagnosis of AA; Intervention—measurement of PTX-3 levels in these patients; Comparison—PTX-3 levels in healthy controls or patients with non-specific abdominal pain (without any clinico-radiologic features of AA); Outcomes—Comparison of PTX-3 levels between cases with AA vs healthy controls, and cases with AA vs cases with non-specific abdominal pain (NSAP) were the main outcomes studied in this review. Secondary outcomes were comparison of PTX-3 levels between perforated vs non-perforated appendicitis, between children with AA vs healthy controls, and children with AA vs children with NSAP. All studies reporting one of the main outcomes were eligible for inclusion. Review articles, case reports, case series, editorials, opinion articles, commentaries, and conference abstracts were also excluded. The studies with unavailable full texts or with incomplete data were also excluded.

### Data synthesis

Two investigators, NK and SA independently extracted the data utilizing Microsoft Excel spreadsheets (Version 15.24). Along with the data on the study outcomes, the baseline information (name of the first author, year of publication, the type of study design, the number of patients in each study, and the number of patients in each patient group) regarding the included studies were recorded. Any disagreements among these authors were resolved through discussion with the third investigator (PG).

### Quality assessment

The methodological quality assessment was independently performed by two investigators (SA and NP) utilizing the Downs and Black scale [8]. This scale has four assessment domains with minimum and maximum scores of 0 and 32, respectively. The risk of bias in the included studies was categorized as high (0–15), moderate (16–23), or low (> 23) on the basis of the scores assigned by each investigator. The inter-observer agreement was calculated using the kappa statistics. Based on the kappa values, the level of agreement was defined as poor (< 0.00), slight (0.00–0.20), fair (0.21–0.40), moderate (0.41–0.60), substantial (0.61–0.80) and almost perfect (0.81–1.00) [9].

### Data analysis

The meta-analysis of the outcomes was performed using RevMan 5.4 (Cochrane Collaboration, London, UK). As all the outcomes were continuous, mean differences (MD) were calculated for each of them. Subsequently, the inverse variance method was used to calculate the weighted mean difference (WMD). The *I*^2^ statistics demonstrated the heterogeneity among the included studies. A random-effects model was chosen if the heterogeneity was substantial (*I*^2^ > 50%). A *p* value of < 0.05 was considered statistically significant.

## Results

### Characteristics of the included studies

**Fig. 1:**
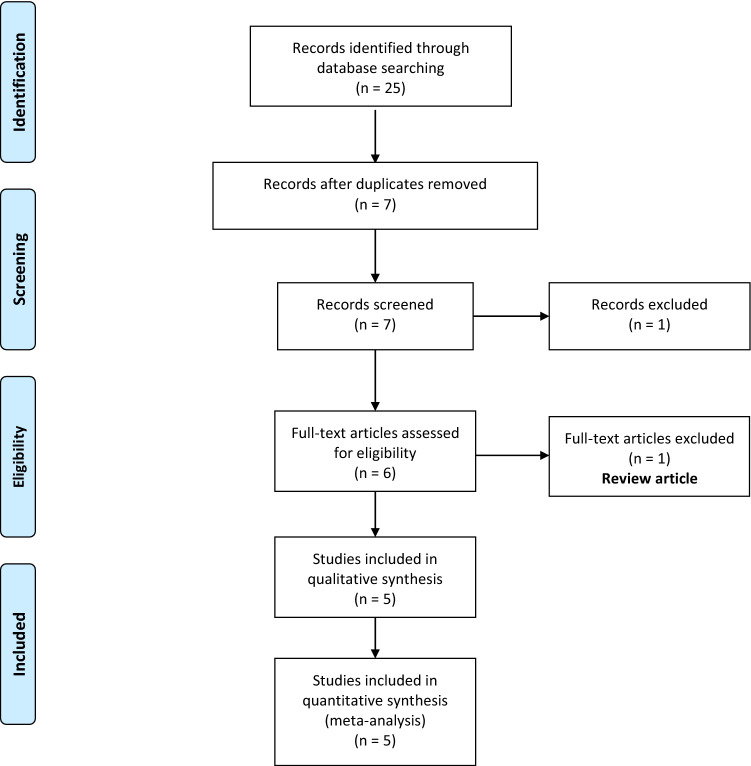
Selection of the relevant studies using the Preferred Reporting Items for Systematic Review and Meta-Analysis (PRISMA) flow diagram

A total of 25 articles were identified with our search strategy (Annexure A). Of these, eighteen were duplicate records and were eliminated (Fig. 1). Out of seven screened abstracts, one was excluded [10]. Only six full-texts were assessed for eligibility. One of them was further excluded as it was a review article [11]. Therefore, only five studies were included in the final meta-analysis [1, 2, 12–14]. All of these were prospective cohort studies. The baseline characteristics of the included studies are depicted in Table 1. Three out of five studies included only children. A male preponderance was noticed among all studies.

**Table 1:** Baseline characteristics of the included studies

Authors	Study design	Sample size			Gender (M:F)			Patient population
		AA	NSAP	HC	AA	NSAP	HC	
Aygun et al. 2019 [12]	Pro	39	12	31	25:14	8:4	21:10	Adults
Oztan et al. 2019 [2]	Pro	34	26	28	22:12	12:14^a^	24:4	Children
Duman et al. 2020 [1]	Pro	37	25	8	26:11	15:10	5:3	Children
Gul et al. 2020 [13]	Pro	112	–	65	59:53	–	35:30	Adults
Ates et al. 2020 [14]	Pro	40	–	15	Groupwise distribution not mentioned			Children

*Pro* prospective cohort study, *AA* acute appendicitis, *NSAP* non-specific abdominal pain, *HC* healthy controls, *M:F* Male:Female

^a^Among the included studies, a male preponderance was noticed in all patient groups except this group

### Methodological quality assessment

The Downs and Black scoring by two independent authors are depicted in Table 2. The average scores ranged from 16.5 to 22. The minimum and maximum scores were assigned to Duman et al. and Oztan et al., respectively. All the studies had a moderate risk of bias. The inter-observer agreement was almost perfect (Kappa = 0.9397, *p* < 0.0001).

**Table 2:** Downs and Black scale scores for the included studies by observer 1 and observer 2. The total scores and inter-observer agreement are also depicted in the table

Study	Reporting	External validity	Internal validity- bias	Internal validity-confounding	Power	Total scores
Methodological assessment by author 1						
Aygun et al. 2019 [12]	8	1	5	3	2	19
Oztan et al. 2019 [2]	7	2	5	3	5	22
Duman et al. 2020 [1]	7	1	5	3	0	16
Gul et al. 2020 [13]	7	1	5	3	5	21
Ates et al. 2020 [14]	8	1	5	3	3	20
Methodological assessment by author 2						
Aygun et al. 2019 [12]	8	1	5	3	2	19
Oztan et al. 2019 [2]	8	1	5	3	5	22
Duman et al. 2020 [1]	8	1	5	3	0	17
Gul et al. 2020 [13]	8	1	5	3	5	22
Ates et al. 2020 [14]	8	1	5	3	3	20

Total scores and inter-observer agreement					
Study	Rater 1	Rater 2	Mean	Kappa value	*p* value
Aygun et al. 2019 [12]	19	19	19	0.9397	< 0.0001
Oztan et al. 2019 [2]	22	22	22		
Duman et al. 2020 [1]	16	17	16.5		
Gul et al. 2020 [13]	21	22	21.5		
Ates et al. 2020 [14]	20	20	20		

### Outcome analysis

**Fig. 2:**
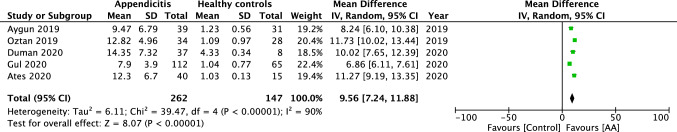
Forest plot comparison of serum Pentraxin-3 levels between the two patient groups, i.e., cases with acute appendicitis vs healthy controls. *AA* acute appendicitis

**Fig. 3:**
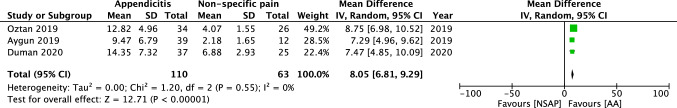
Forest plot comparison of serum Pentraxin-3 levels between the two patient groups, i.e., cases with acute appendicitis vs non-specific abdominal pain. *AA* acute appendicitis, *NSAP* non-specific abdominal pain

**Fig. 4:**
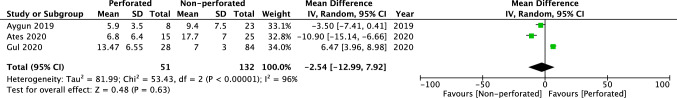
Forest plot comparison of serum Pentraxin-3 levels between the two patient groups, i.e., cases with perforated vs non-perforated appendicitis

**Fig. 5:**
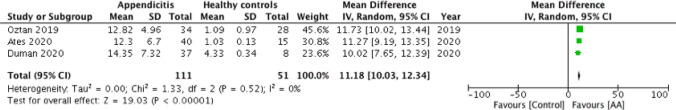
Forest plot comparison of serum Pentraxin-3 levels between the two patient groups, i.e., children with acute appendicitis vs healthy controls. *AA* acute appendicitis

**Fig. 6:**
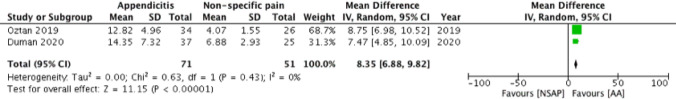
Forest plot comparison of serum Pentraxin-3 levels between the two patient groups, i.e., children with acute appendicitis vs non-specific abdominal pain. *AA* acute appendicitis, *NSAP* non-specific abdominal pain

- **Comparison of PTX3 levels among cases with AA **vs** healthy controls**
  This outcome was reported by all five studies [1, 2, 12–14]. PTX3 levels were compared among 262 cases with AA vs 147 healthy controls. Pooling the data (Fig. 2) demonstrated significantly higher levels of PTX3 in cases with AA vs healthy controls (WMD: 9.56, 95% CI 7.24–11.88, *p* < 0.00001). For this outcome, the estimated heterogeneity among the included studies was substantial and statistically significant (*I*^2^ = 90%, *p* < 0.00001).
- **Comparison of PTX3 levels among cases with AA **vs** NSAP**
  Three studies, including 110 and 63 cases within the AA and NSAP patient groups, respectively, reported this outcome [1, 2, 12]. Pooling the data (Fig. 3) demonstrated significantly higher levels of PTX3 in cases with AA vs NSAP (WMD: 8.05, 95% CI 6.81–9.29, *p* < 0.00001). For this outcome, the estimated heterogeneity among the included studies was neither substantial and nor statistically significant (*I*^2^ = 0%, *p* = 0.55).
- **Comparison of PTX3 levels among perforated **vs** non-perforated appendicitis**
  Four studies depicted the distribution of complicated and non-complicated AA [1, 12–14]. However, the actual PTX3 levels of complicated and non-complicated groups were not reported by Duman et al. [1]. Only three studies [12–14] clearly compared the levels of PTX3, that too between two specific patient groups, i.e., perforated vs non-perforated. The comparison included 51 cases with perforated appendicitis vs 132 cases with non-perforated appendicitis. Pooling the data (Fig. 4) demonstrated no significant difference among these patient groups in terms of PTX3 levels (WMD: – 2.54, 95% CI – 12.99 to 7.92, *p* = 0.63). The estimated heterogeneity among the included studies was substantial and statistically significant (*I*^2^ = 96%, *p* < 0.00001) for this outcome.
- **Comparison of PTX3 levels among children with AA **vs** healthy controls**
  Three studies, including 111 children with AA and 51 healthy controls, respectively, reported this outcome [1, 2, 14]. Pooling the data (Fig. 5) demonstrated significantly higher levels of PTX3 in children with AA vs healthy controls (WMD: 11.18, 95% CI 10.03–12.34, *p* < 0.00001). For this outcome, the estimated heterogeneity among the included studies was neither substantial and nor statistically significant (*I*^2^ = 0%, *p* = 0.52).
- **Comparison of PTX3 levels among children with AA **vs** NSAP**
  This outcome was reported by two studies only [1, 2]. PTX3 levels were compared among 71 children with AA vs 51 children with NSAP. Pooling the data (Fig. 6) demonstrated significantly higher levels of PTX3 in children with AA vs NSAP (WMD: 8.35, 95% CI 6.88–9.82, *p* < 0.00001). For this outcome, the estimated heterogeneity among the included studies was neither substantial and nor statistically significant (*I*^2^ = 0%, *p* = 0.43).

## Discussion

AA still presents a diagnostic challenge, especially in younger children. Any delay in diagnosis may entail a complicated course with prolonged treatment. The available literature highlights that PTX3 levels rise as early as 6 h after the onset of inflammatory processes [5]. We demonstrate here, that PTX3 levels can discriminate between cases with AA vs NSAP or healthy controls, making it an ideal candidate for early identification of AA. However, this biomarker does not indicate further details regarding the clinical course of AA.

Pentraxins are a conserved family of proteins of different lengths, involved in various cellular activities ranging from immediate inflammatory responses to pathogen modulation [3]. The short pentraxin CRP has traditionally been utilized as an acute-phase protein to indicate bacterial infection, but it is limited by a delayed response [4]. PTX3 represents a longer pentraxin and shows promise with a significantly earlier response than CRP, thus, identifying inflammatory responses acutely. While the C-terminal region of PTX3 is homologous to CRP, the N-terminal region is unique and presumably involved in binding activities [15]. The binding activities of PTX3 allow for a multitude of functions with infection control, immune modulation and complement activation [16].

Earlier studies have shown promise of PTX3 in AA diagnostics with improved sensitivity and specificity compared to previous parameters, but to our knowledge, our report is the first systematic review and meta-analysis on this subject. We show that the sensitivity of PTX3 in AA is good. Two of the included studies addressed PTX3 in AA vs healthy controls (or cases with NSAP) in adults while three studies depicted this in children. In our analysis, both of these cohorts demonstrated elevated PTX3 levels in AA. Nevertheless, PTX3 does not seem to distinguish between perforated and non-perforated AA, as the analysis of the pooled data did not yield a statistically significant rise in PTX3 levels among patients with perforated appendicitis. The exact reason behind this finding is not well-understood. In addition, significantly higher levels of other acute-phase reactants in cases with perforated vs non-perforated appendicitis make this issue even more complicated [13]. One of the possible explanations for this can be based on the practice of early introduction of antibiotics in these cases. PTX3 has a short half-life of 1–4 h [17]. Early antibiotic introduction can decrease the quantum of release of PTX3 in the systemic circulation; however, the local inflammatory responses continue, ultimately leading to appendicular perforation. Therefore, as per the current literature, serum PTX3 is not an ideal biomarker for differentiating between these two patient groups. On the other hand, Alvarez-Alvarez et al. have shown that serum fibrinogen can accurately predict appendicular perforation with a sensitivity and specificity of 87 and 91%, respectively, thus, making it the biomarker of interest for future studies [18].

AA is the most common abdominal surgical emergency among children, yet the exact pathophysiology of AA remains unclear [19]. While common, AA can still present a diagnostic challenge to clinicians. The diagnosis is a combination of careful history, clinical findings, diagnostic tests and different imaging modalities. Several appendicitis scoring systems have been introduced over the years. A substantial proportion of patients with right lower quadrant pain do not suffer from acute appendicitis and non-operative treatment of uncomplicated acute appendicitis is gaining popularity [20, 21]. Therefore, it is of paramount importance to make a correct diagnosis at the time of emergency referral. While PTX3 shows promise in early diagnosis for AA, the present meta-analysis reiterates that clinical vigilance remains essential to identify a complicated course timely.

It is also noteworthy that the term ‘complicated appendicitis’ also includes other pathological entities and is not just perforated appendicitis. The current literature is limited and has studies focusing on the ability of PTX3 to discriminate between perforated vs non-perforated AA only. Due to this limitation, the results of the present meta-analysis should not be extrapolated to broader comparison groups, i.e., complicated vs non-complicated AA. Further prospective comparative studies are needed to explore whether this inability of PTX3 to differentiate between a complicated vs an uncomplicated course is due to a restricted patient selection criteria or a true inability to differentiate between these inflammatory pathologies.

The results of the present study must be interpreted within the context of a few limitations. First, all the included comparative studies have a moderate risk of bias. Second, the sample size of the available studies is also limited. Third, the meta-analysis includes both children and adults, possibly confounding the results. Although we performed a separate meta-analysis including only children, a more homogeneous patient cohort need to be studied in future studies. Fourth, being a non-specific marker of inflammation, PTX3 levels tend to rise in all inflammatory conditions. Fifth, as mentioned above, broader comparison groups, i.e., complicated and non-complicated AA need to be studied in future studies. Therefore, the baseline characteristics of the patients need to be studied in more detail to exclude the possibility of concurrent inflammatory conditions. Finally, rather than focusing on the sensitivity and specificity of a single biomarker, subsequent studies need to explore the accuracy of a panel of biomarkers. The panel must include biomarkers that have specific roles (either diagnostic or prognostic) and must complement each other. Based on the findings of this study and the information provided by the available literature, it will be interesting to explore the accuracy of a panel of biomarkers including serum PTX3, serum fibrinogen, total leucocyte count, and neutrophil-to-lymphocyte (NLR) ratio.

## Conclusions

When compared to healthy controls and cases with NSAP, patients with AA have significantly higher levels of PTX3. This elevation of serum PTX3 is observed in both children and adults with AA. However, this biomarker cannot differentiate between perforated and non-perforated appendicitis. Due to the moderate risk of bias of the available comparative studies, adequately powered prospective studies need to be conducted before any definite conclusions are drawn.

## References

[1] Duman L, Cesur Ö, KumbulDoğuç D (2020). Diagnostic value of serum pentraxin 3 level in children with acute appendicitis. Ulus Travma Acil Cerrahi Derg.

[2] Oztan MO, Aksoy Gokmen A, Ozdemir T (2019). Pentraxin-3: a strong novel biochemical marker for appendicitis in children. Am J Emerg Med.

[3] Doni A, Stravalaci M, Inforzato A (2019). The long Pentraxin PTX3 as a link between innate immunity, tissue remodeling, and cancer. Front Immunol.

[4] Peri G, Introna M, Corradi D (2000). PTX3, a prototypical long pentraxin, is an early indicator of acute myocardial infarction in humans. Circulation.

[5] Jaillon S, Peri G, Delneste Y (2007). The humoral pattern recognition receptor PTX3 is stored in neutrophil granules and localizes in extracellular traps. J Exp Med.

[6] Cieslik P, Hrycek A (2012). Long Pentraxin 3 (PTX3) in the light of its structure, mechanism of action and clinical implications. Autoimmunity.

[7] Moher D, Liberati A, Tetzlaff J (2009). Preferred reporting items for systematic reviews and meta-analyses: the PRISMA statement. BMJ.

[8] Downs SH, Black N (1998). The feasibility of creating a checklist for the assessment of the methodological quality both of randomised and non-randomised studies of health care interventions. J Epidemiol Community Health.

[9] Landis JR, Koch GG (1977). The measurement of observer agreement for categorical data. Biometrics.

[10] Chisthi M (2019). Brief commentary on the article "diagnostic value of plasma Pentraxin-3 in acute appendicitis". J Invest Surg.

[11] Abouhamda A (2020). Pentraxin-3, Interleukin-6, and acute appendicitis: biomarkers that need further exploration. Cureus.

[12] Aygun A, Katipoglu B, Ïmamoglu M (2019). Diagnostic value of plasma Pentraxin-3 in acute appendicitis. J Invest Surg.

[13] Gul VO, Destek S (2020). Using Pentraxin-3 for diagnosing acute appendicitis and predicting perforation: a prospective comparative methodological study. Ulus Travma Acil Cerrahi Derg.

[14] Ates U, Bahadir K, Ergun E (2020). Determination of pentraxin 3 levels in diagnosis of appendicitis in children. Pediatr Int.

[15] Bottazzi B, Doni A, Garlanda C (2010). An integrated view of humoral innate immunity: pentraxins as a paradigm. Annu Rev Immunol.

[16] Inforzato A, Bottazzi B, Garlanda C (2012). Pentraxins in humoral innate immunity. Adv Exp Med Biol.

[17] Albert Vega C, Mommert M, Boccard M (2019). Source of circulating Pentraxin 3 in septic shock patients. Front Immunol.

[18] Alvarez-Alvarez FA, Maciel-Gutierrez VM, Rocha-Muñoz AD (2016). Diagnostic value of serum fibrinogen as a predictive factor for complicated appendicitis (perforated). A cross-sectional study Int J Surg.

[19] Bhangu A, Soreide K, di Saverio S (2015). Acute appendicitis: modern understanding of pathogenesis, diagnosis and management. Lancet.

[20] Salminen P, Tuominen R, Paajanen H (2018). Five-year follow-up of antibiotic therapy of uncomplicated acute appendicitis in the APPAC randomized clinical trial. JAMA.

[21] Minneci PC, Mahida JB, Lodwick DL (2016). Effectiveness of patient choice in nonoperative vs surgical management of pediatric uncomplicated acute appendicitis. JAMA Surg.

